# OA23 Setting up a Young Persons Rheumatology Clinic. Sharing our learning from Central Lancashire

**DOI:** 10.1093/rap/rkag113.023

**Published:** 2026-11-03

**Authors:** Elizabeth MacPhie, Eleanor Heaf

**Affiliations:** Lancashire and South Cumbria NHS Foundation Trust, Preston, United Kingdom; Lancashire Teaching Hospitals NHS Foundation Trust, Preston, United Kingdom

## Abstract

**Introduction:**

Transition care from paediatrics to an adult clinic is crucial for improving long-term health outcomes by empowering young people to manage their conditions, reduce the risk of loss to follow-up and treatment being interrupted, and ensuring continuity of care during a significant life change. The community-based adult rheumatology service in Preston recognised that many young people (YP) were being lost after being referred from paediatric rheumatology clinics. The opportunity was identified to set up a Young Persons Clinic with the paediatric rheumatologist based at the acute trust to enable a smoother transition.

**Case description:**

Managers acknowledged that a joint clinic where YP could be seen by both their known paediatric consultant and the adult consultant who would be taking over their care avoided duplication. No business case was needed as there was no additional funding required and estates were available. The clinic is delivered from the community setting where the adult service is based. Appointments are 45 minutes for new and 30 minutes for follow-up. There is time assigned at the start of clinic for discussion about the patients attending that day. YP turning 16yrs of age are identified and put forward for the next clinic. An appointment letter explaining the purpose of the clinic is sent and a text reminder the week before. YP are seen as many times as is necessary until they are ready to transfer to the adult clinic. There are clear instructions to all regarding the handover of prescribing of disease modifying and high-cost drugs. Drug education is revisited to ensure YP are reminded of key messaging, in addition to the practicalities of prescriptions and monitoring.

There have been 6 clinics over the 2 years at 6-monthly intervals and 15 patients have been seen. 12 of the patients were female and 6 of the patient were under 10 years of age. when diagnosis. 22 appointments have been offered (17 new, 5 follow-up) and 2 YP failed to attend. Diagnoses of those seen: JIA (n = 9), CTD (n = 5), Behcets (n = 1.) 10 YP were transferred to the adult clinic after one appointment, 3 required two appointments before handover of care and one had three appointments. 9 of the patients have transferred over on a DMARD or high cost drug, 4 on combination therapy and 4 on infusions.

**Discussion:**

There has been considerable learning from setting up this clinic. Timing of clinics is imperative and the need to avoid exams periods and school holidays, March and October have been identified as optimum months. In addition to the appointment letter and text reminders we also phone the week of the clinic.

We structure the appointment so that we focus first on education and how school or college is going. We recognise this helps the YP to relax and build a rapport and understand that we see them as a person not a patient.

We recognise the importance of discussions around medications and the handover of prescribing. An update around drug education is essential recognising that many started treatment years previously and there will be other factors to consider which may not have previously been discussed. The practicalities of how monitoring and prescribing is undertaken in an adult clinic is a big change for our YP and we have become alert to issues around compliance and stockpiling of medication. Many of the YP have needle phobias and for the adult rheumatology team we have had to understand how we support our YP as this is not something we have been used to dealing with.

If patients are continuing on treatment with a DMARD or high-cost drug we now invite one of the specialist nurses to join the appointment so the YP can meet the nurse they will be seeing for drug education. If patients are on an infusion we show them the infusion room and introduce them to the clinic nurses. Communicating with our YP has been a challenge and we encourage them to have their mobile number as the main contact for text reminders rather than their parents. Next steps are to involve YP in the evaluation of the clinic.

**Key learning points:**

• Setting out on this venture we were concerned about the red tape and that there may be a requirement for a business case. We were very aware that there was no funding available. Onboarding managers and supporting them to see the benefits of the clinic was imperative. After every clinic we have made changes and it has been really important to reflect each time on what has gone well and what could we do better. Input from the rest of the rheumatology multi-disciplinary team has been hugely beneficial and led to additional changes to how we run the clinic. Every clinic is a fantastic learning opportunity for both the paediatric and adult rheumatologist. We recognise that not every hospital will be able to set up a clinic in the same way we have done but we hope to enthuse and share our learning and help others navigate the red tape.

